# Oncological and Surgical Outcomes of Left Trisegmentectomy or Lingulectomy Versus Upper Lobectomy for Early-Stage Non-Small Cell Lung Cancer: A Multicentre Study

**DOI:** 10.1093/ejcts/ezaf427

**Published:** 2025-11-26

**Authors:** Dania Nachira, Maria Teresa Congedo, Giuseppe Calabrese, Alessia Senatore, Khrystyna Kuzmych, Gloria Santoro, Beatrice Trabalza Marinucci, Giorgia Piccioni, Michele Salati, Anna Chiara Nanto, Riccardo Orlandi, Mirko Girolamo Cantatore, Rosalia Romano, Sara Ricciardi, Andrea Imperatori, Pietro Bertoglio, Angela De Palma, Mohsen Ibrahim, Majed Refai, Paolo Mendogni, Diego Gavezzoli, Giuseppe Cardillo, Giuseppe Marulli, Piergiorgio Solli, Elisa Meacci, Stefano Margaritora

**Affiliations:** Department of General Thoracic Surgery, Fondazione Policlinico Universitario “A. Gemelli”, IRCCS, Università Cattolica del Sacro Cuore, 00168 Rome, Italy; Department of General Thoracic Surgery, Fondazione Policlinico Universitario “A. Gemelli”, IRCCS, Università Cattolica del Sacro Cuore, 00168 Rome, Italy; Department of General Thoracic Surgery, Fondazione Policlinico Universitario “A. Gemelli”, IRCCS, Università Cattolica del Sacro Cuore, 00168 Rome, Italy; Department of General Thoracic Surgery, Fondazione Policlinico Universitario “A. Gemelli”, IRCCS, Università Cattolica del Sacro Cuore, 00168 Rome, Italy; Department of General Thoracic Surgery, Fondazione Policlinico Universitario “A. Gemelli”, IRCCS, Università Cattolica del Sacro Cuore, 00168 Rome, Italy; Department of Medical and Surgical Sciences, Fondazione Policlinico Universitario “A. Gemelli”, IRCCS, Università Cattolica del Sacro Cuore, 00168 Rome, Italy; Division of Thoracic Surgery, Sant’Andrea Hospital, Sapienza University, Rome, 00189, Italy; Division of Thoracic Surgery, Sant’Andrea Hospital, Sapienza University, Rome, 00189, Italy; Unit of Thoracic Surgery, AOU of Marche, 60126 Ancona, Italy; Unit of Thoracic Surgery, AOU of Marche, 60126 Ancona, Italy; Thoracic Surgery and Lung Transplantation—IRCCS Foundation Ca’ Granda Ospedale Maggiore Policlinico, Milan, 20122, Italy; Unit of Thoracic Surgery, Department of Precision and Regenerative Medicine and Ionian Area, University of Bari “Aldo Moro”, 70124 Bari, Italy; Division of Thoracic Surgery, IRCCS Sacro Cuore-Don Calabria Hospital, Negrar di Valpolicella, Verona, 37024, Italy; Unit of Thoracic Surgery, Azienda Ospedaliera San Camillo Forlanini, 00152 Rome, Italy; Research Center of Minimally Invasive Surgery and Thoracic Surgery, Department of Medicine and Technological Innovation (Department of Medicine and Surgery), University of Insubria, Varese, 21100, Italy; Division of Thoracic Surgery, IRCSS Azienda Ospedaliero-Universitaria, Bologna, 40138, Italy; Unit of Thoracic Surgery, Department of Precision and Regenerative Medicine and Ionian Area, University of Bari “Aldo Moro”, 70124 Bari, Italy; Division of Thoracic Surgery, Sant’Andrea Hospital, Sapienza University, Rome, 00189, Italy; Unit of Thoracic Surgery, AOU of Marche, 60126 Ancona, Italy; Thoracic Surgery and Lung Transplantation—IRCCS Foundation Ca’ Granda Ospedale Maggiore Policlinico, Milan, 20122, Italy; Division of Thoracic Surgery, IRCCS Sacro Cuore-Don Calabria Hospital, Negrar di Valpolicella, Verona, 37024, Italy; Unit of Thoracic Surgery, Azienda Ospedaliera San Camillo Forlanini, 00152 Rome, Italy; Division of Thoracic Surgery, IRCCS Humanitas Research Hospital, 20089 Rozzano, Milan, 20089, Italy; Thoracic Surgery Unit, Fondazione IRCCS Istituto Nazionale Tumori, 20131 Milan, Italy; Department of General Thoracic Surgery, Fondazione Policlinico Universitario “A. Gemelli”, IRCCS, Università Cattolica del Sacro Cuore, 00168 Rome, Italy; Department of General Thoracic Surgery, Fondazione Policlinico Universitario “A. Gemelli”, IRCCS, Università Cattolica del Sacro Cuore, 00168 Rome, Italy

**Keywords:** NSCLC, trisegmentectomy, lingulectomy, lobectomy

## Abstract

**Objectives:**

Left upper lobe and right upper and middle lobes have a similar anatomical structure; therefore, multi-segmentectomy (S), as upper trisegmentectomy and lingulectomy, should guarantee the same oncological radicality as left upper lobectomy (LUL). The aim of the study was to compare the oncological and surgical outcomes of left upper S (trisegmentectomy or lingulectomy) and LUL for early-stage (cT1-T2bN0M0) non-small cell lung cancer (NSCLC).

**Methods:**

Clinical data of patients who underwent S or LUL, without any previous neoadjuvant treatment, from June 2016 to March 2024 at 9 high-volume centres, were retrospectively reviewed. To reduce any selection bias, a 1:1 propensity score matching (PSM) was performed.

**Results:**

After PSM, 105 patients, with comparable clinical-pathological characteristics, were included in each group. A significant difference between LUL and S was recorded in terms of postoperative complications (*P* = .014), mainly air-leak (*P* = .055), without differences in hospitalization (*P* = .333). The median follow-up (FUP) time was 23 months (interquartile range [IQR]: 13-42). The 5-year overall survival (OS) and disease-free survival (DFS) were 95% vs 80% (*P* = .072) and 97%vs 83%(*P* = .090) for LUL and S, respectively. When analysing only cases with tumour-to-margin distance of <1 cm, the 5-year OS and DFS were LUL: 98% vs S: 64%(*P* = .049) and LUL: 96% vs S: 87%(*P* = .056), respectively. If the distance was > 1 cm, the 5-year OS (*P* = .193) and DFS (*P* = .351) were comparable in both groups, as for tumour-diameter > 2 cm (5-year OS (*P* = .429) and DFS (*P* = .602)). In the S cohort, no difference was found between lingulectomy and trisegmentectomy in terms of 5-year OS (*P* = .240) and DFS (*P* = .304). At multivariable analysis, positive spread-through air spaces (STAS) was the only significant predictor for DFS in S group (*P* = .027).

**Conclusions:**

Oncological outcomes of left upper trisegmentectomy or lingulectomy for early-stage NSCLC are comparable with those of LUL, if the tumour is located more than 1 cm from the surgical margin. Positive STAS is the only predictor for shorter DFS in S group.

## GRAPHICAL ABSTRACT

**Figure:**
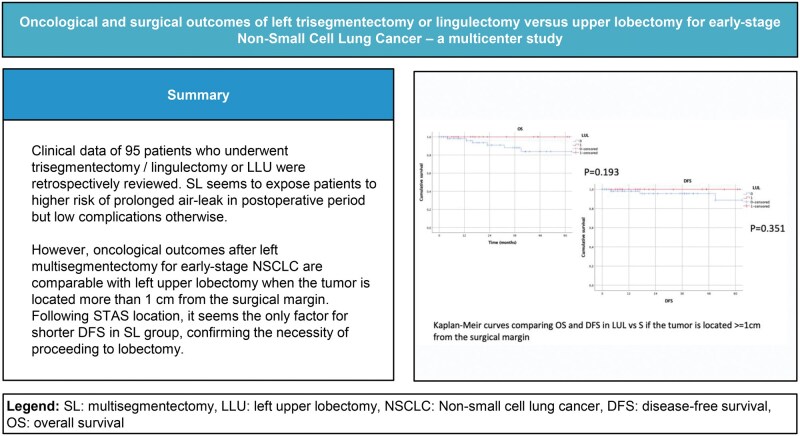

## INTRODUCTION

With the advancement of mini-invasive technique and the increasing number of diagnosis of early-stage lung cancer, lung-sparing resections are more frequent than in the past. In particular, the number of pulmonary segmentectomies and multi-segmentectomies (S) has been progressively growing, from an average of 3.3% per year in 2004 to 6.1% in 2018,^1^ according to the US National Cancer Database.

The widespread adoption of this technique was limited by the fact that, for a long time, segmentectomies were reserved as an alternative to lobectomy in patients at high risk of respiratory failure or deemed unfit for radical excision. Moreover, segmentectomy was considered technically demanding, particularly when performed using robotic or video-assisted approaches, as it was associated with a higher risk of postoperative complications^2^—especially prolonged air leaks—and an increased rate of local recurrence.

Since the publication of the results of the multi-institutional Japanese randomized JCOG0802/WJOG4607 trial,^3^ which reported a superiority of segmentectomy over lobectomy in terms of OS for tumour diameter ≤2 cm and consolidation-to-tumour ratio >0.5, the number of segmentectomies performed have increased rapidly.

Regarding the treatment of ground-glass opacities (GGOs) and part-solid nodules, the recent European Respiratory Society/European Society of Thoracic Surgeon (ERS/ESTS) guidelines^4^ recommended to perform a wedge resection in peripheral pure-GGO and a segmentectomy in central pure-GGO, while an anatomical resection, like a segmentectomy, is recommended for part-solid nodule.

In a recent comparative study,^5^ oncologic surgical procedures of lobectomy and limited resection yielded comparable outcomes in patients with clinical stage I GGO-dominant lung adenocarcinomas ≤2 cm, while lobectomy showed better survival compared to limited resection in patients with solid-dominant tumour.

Few evidence still exists on the role of S for treatment of solid lesions of ≤2 cm. In this scenario, the left apical trisegmentectomy (S1-2+S3) and the lingulectomy (S4+S5)—having the same anatomical structure as right upper lobe (S1+S2+S3) and middle lobe (S4+S5), with autonomous vascular hilar structures and bronchi—are considered to be equivalent to a lobectomy^6^^,^^7^ and some studies have reported similar oncological outcomes between trisegmentectomy and lingulectomy vs left upper lobe.^8–12^

Therefore, the main aim of the study was to compare the oncological and surgical outcomes of left upper S (trisegmentectomy or lingulectomy) and left upper lobectomy (LUL) for early-stage (cT1-T2bN0M0) non-small cell lung cancer (NSCLC).

## METHODS

### Study design

The present study is a retrospective, multicentre study, approved by the Ethical Committee (Università Cattolica del Sacro Cuore, Approval Code ID: 3553) and therefore conducted in accordance with the ethical standards of the Declaration of Helsinki and its later amendments. All patients provided informed consent for inclusion in the study.

Data and results are reported according to the Strengthening the Reporting of Observational Studies (STROBE) checklist.

From June 2016 to March 2024, clinical data of 538 patients who underwent multiple upper left S (trisegmentectomy or lingulectromy) or LUL for early stage (cT1-T2bN0M0) NSCLC (TNM Eighth Edition) at 9 Italian high-volume centres were retrospectively reviewed.

**Figure 1.**
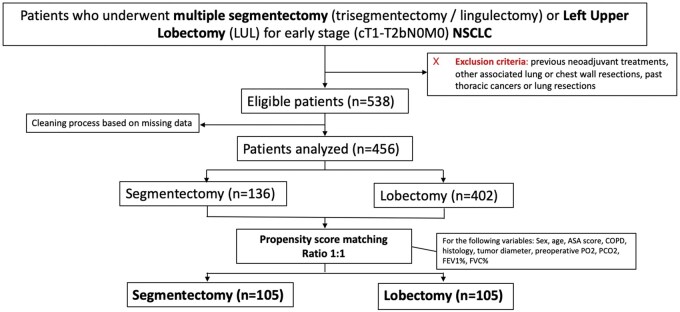
Flowchart of the Selection Process of the Study. Abbreviations: LUL: left upper lobe; NSCLC: non-small cell lung cancer

The exclusion criteria were: previous neoadjuvant treatments, other associated lung or chest wall resections, past thoracic cancers or lung resections (**Figure 1**).

All patients underwent routine preoperative examinations (including lung functionality tests) and staging. The choice between LUL or S was determined by the surgeons of each centre involved based on patient’s clinical conditions and preoperative evaluation, tumour location, and dimension.

Surgery was performed under general anaesthesia and single-lung ventilation.

Each centre adopted the preferred approach in which it had extensive expertise among: video-assisted thoracic surgery (uniportal or multiportal VATS), robotic-assisted thoracic surgery (RATS), or lateral thoracotomy. Therefore, surgical performance can reasonably be expected to be of the highest standard.

Tumour-to-margin distance (pulmonary fissure or intersegmental plane) was measured macroscopically by the pathologist on surgical specimen during definitive histological examination (following formalin fixation of the specimen, with consideration of tissue shrinkage).

As a standard practice, surgical margins were not consistently assessed on fresh frozen sections after segmentectomy; instead, their adequacy was systematically evaluated grossly by the surgeon during the procedure. Fresh frozen section on intersegmental lymph nodes was not performed.

Data regarding postoperative follow-up (FUP) were obtained from radiological examinations (total-body computed tomography [CT] scan and 18 F-fluorodeoxyglucose positron emission tomography [PET] scan) and periodical oncological evaluations of the patients performed at least for the first 5 years after surgery. The overall survival (OS) was defined as the time elapsed from the date of surgery and the date of death for disease. The disease-free survival (DFS) as the time elapsed from the date of surgery and the date of local or distance recurrence. Right censoring occurred when the event happened after the FUP period of the study.

The primary outcome of the study was to evaluate oncological and surgical outcomes of left upper S compared to LUL for the treatment of early stage (cT1-T2bN0M0) NSCLC.

The secondary outcomes were to evaluate in the cohort of S any differences in oncological outcomes between lingulectomy and trisegmentectomy.

### Statistical analysis

Continuous variables were expressed as mean ± SD or medians and interquartile range (IQR) if not normally distributed, while categorical variables were as numbers and percentages. Continuous variables were compared using the independent-sample Student’s t-test or the Mann-Whitney U-test if normally or non-normally distributed (according to the Shapiro-Wilk test). Categorical variables were compared by Chi-square test.

A cleaning process was performed, eliminating all patients with missing data > 25% for the 118 variables evaluated in the dataset, obtaining only patients with complete data for the 69 variables considered fundamental for the analysis (**Figure 1**).

Baseline clinical characteristics were compared between the 2 groups (S and LUL) using the methods described above (**Table 1**).

**Table 1.** Patient Clinical Characteristics Before and After PSM (in Bold Significant Values < 0.05)

	Unmatched groups			Matched groups		
	LUL (*n* = 402)	Segmentectomy (*n* = 136)	*P*	LUL (*n* = 105)	Segmentectomy (*n* = 105)	*P*
Male sex	214 (53.23%)	76 (55.88%)	.592	50 (47.61%)	60 (57.14%)	.167
ASA score	2.61 ± 0.81	2.26 ± 0.60	**<.001**	3.30 ± 0.68	3.10 ± 1.02	.983
Age (years)	66.24 ± 10.06	68.91 ± 9.46	**.004**	67.38 ± 11.49	69.35 ± 9.89	.741
COPD	100 (24.88%)	41 (30.15%)	.145	34 (32.38%)	34 (32.38%)	1.00
Smoking	179 (44.53%)	31 (22.79%)	**<.001**	82 (78.09%)	68 (64.76%)	.109
Histology (adenocarcinoma)	132 (32.84%)	113 (83.08%)	**<.001**	90 (85.71%)	95 (90.48%)	.120
Tumour dimension (cm)	1.67 ± 0.69	1.65 ± 0.71	.772	1.51 ± 0.53	1.67 ± 0.67	.103
Consolidation-to-tumour ratio =1	361 (89.8%)	105 (77.2%)	**<.001**	87 (82.9%)	76 (72.4%)	.178
Preoperative PO_2_	78.41 ± 28.09	90.13 ± 19.79	**<.001**	86.15 ± 19.55	86.36 ± 9.66	.920
Preoperative PCO_2_	49.74 ± 20.54	47.39 ± 19.37	**.002**	40.45 ± 7.01	41.01 ± 4.44	.940
Preoperative FEV1%	93.44 ± 23.48	90.77 ± 21.39	.315	95.26 ± 21.62	92.53 ± 18.00	.320
Preoperative FVC%	100.26 ± 22.70	99.50 ± 22.28	.783	100.37 ± 22.25	100.64 ± 19.33	.930

Abbreviations: ASA: American Society of Anesthesiology; COPD: chronic obstructive pulmonary disease; LUL: left upper lobectomy; PSM: propensity score matching.

To overcome any potential data biases of a retrospective study, a 1:1 propensity score match (PSC) analysis was performed to balance the baseline characteristics of patients undergone LUL or S. Based on clinical and scientific knowledge, the following variables—potentially associated with the choice of the anatomical lung resection, with outcomes, or with both—were included in the calculation of the propensity score matching (PSM) using the following regression models: sex, age, ASA (American Society of Anesthesiology) score, chronic obstructive pulmonary disease (COPD), histology, tumour diameter, preoperative PCO_2_ (Partial Pressure of Carbon Dioxide), PO_2 _(Partial Pressure of Oxygen), FEV1% (Forced Expiratory volume in 1 second as percentage of the predictive value), and FVC% (Forecd Expiratory Volume in 1 second as a percentage of the Forced Vital Capacity).

Subsequently, the nearest neighbour method was used to find matched pairs, with a calliper width of 0.1 and a distance “logit.” The balance in covariates after matching was assessed by a t-test not significant at 10% (**Table 1**).

Differences in postoperative outcomes (**Table 2**) between the 2 matched groups (LUL and S) were evaluated using the tests for continuous and categorical variables described before.

**Table 2.** Postoperative Outcomes in Matched Groups (in Bold Significant Values < 0.05)

	**LUL** **(*n* = 105)**	**Segmentectomy** **(*n* = 105)**	*P*
Postoperative complications	4 (3.8%)	14 (13.3%)	**.014**
Air-leak >5 days	1 (1%)	6 (5.7%)	**.055**
Atrial fibrillation	1 (0.9%)	2 (1.9%)	.561
Bleeding	0	2 (1.9%)	.155
Subcutaneous emphysema	2 (1.9%)	2 (1.9%)	1.000
Pneumonia	1 (0.9%)	1 (0.9%)	1.000
Hospitalization (days)	4.23 ± 1.22	4.04 ± 1.60	.333
Total LNF retrieved	5.22 ± 2.86	5.79 ± 4.05	.239
Total N1 LNF retrieved	2.85 ± 2.15	2.61 ± 2.61	.471
Total N2 LNF retrieved	2.37 ± 1.53	3.14 ± 2.67	**.011**
Nodal upstaging	5 (4.8%)	10 (9.5%)	.180
Recurrences of disease	2 (1.9%)	8 (7.6%)	**.041**

Abbreviation: LUL: left upper lobectomy; LNF: lymphnodes.

Survival function was estimated by Kaplan-Meier curves for the matched population and comparison of survivals stratified for different clinical categorical variables was performed by log-rank test. Despite the status variable was complete for all matched patients, in 7 cases in S group (all alive without disease), the FUP date was not correct or in invalid format.

Univariable analysis was performed using the Cox regression model. Any variable with a *P*-value less than .20 at univariable analysis was included in a Cox proportional hazards regression model to investigate the adjusted effect of an independent variable on OS and DFS.

A sub-analysis was also performed in the S cohort, performing the same survival and multivariable analyses described above for comparing OS and DFS between trisegmentectomy and lingulectomy and for evaluating any independent variables affecting OS and DFS.

A *P* < .05 was considered statistically significant.

Statistical analysis was performed using the IBM SPSS Statistics for Macintosh, Version 25.00 (Armonk, NY, USA).

## RESULTS

### Overall cohort

After a cleaning process based on missing data, from 538 patients eligible for the study, only 456 patients were considered for the final analysis (**Figure 1**). Among these patients, 402 underwent a LUL, while 136 a S (trisegmentectomy or lingulectomy). Baseline clinical characteristics were not comparable between LUL and S (**Table 1**) due to significant differences in age, ASA score, smoking, histology and preoperative PO_2_ and PCO_2_.

After a PSM, 105 patients were obtained per each group (S and LUL) with comparable baseline characteristics (**Table 1**).

When comparing surgical outcomes between the 2 groups, a significant higher number of postoperative complications was recorded in S group (14 (13.3%) vs 4 (3.8%), *P* = .014), mainly prolonged air-leak > 5 days (6 (5.7%) vs 1 (1%), *P* = .055), without differences in hospital stay (S: 4.04 ± 1.60 vs LUL: 4.23 ± 1.22 days, *P* = .333), **Table 2**.

Although a significant higher number of lymph nodes were retrieved in N2 stations after S (*P* = .011, **Table 2**), no difference in nodal upstaging was recorded between the 2 groups (LUL: 4.8% vs S: 9.5%, *P* = .180).

Thirty- and 90-day mortality were null in both groups.

Recurrence rate was higher in the S group compared to LUL (8 (7.6%) vs 2 (1.9%), *P* = .041).

In the S group, 3 cases presented local recurrence: 2 on surgical margin and mediastinal nodes (both cases were pT1bN0M0 and spread-through air spaces [STAS]-positive), 1 case on ipsilateral visceral and parietal pleura (the pathological stage was pT1bN0M0); while in 5 cases (one STAS-positive), a distant recurrence was recorded (on contralateral lung, bones, brain, adrenal glands, or liver). No patient with a postoperative diagnosis of a STAS-positive tumour in the S group underwent a completion lobectomy but only a strict FUP.

In all cases, recurrence was registered after a median time of 16 months; the tumour was solid with median diameter of 1.5 cm and a distance from margin of 1.5 cm in median. Two patients underwent chemotherapy for the recurrence, 4 patients immunotherapy and radiotherapy, 1 case only immunotherapy, and 1 target-therapy (anti-EGFR drug) + radiotherapy.

In the LUL group, 2 cases (both pT1bN0M0) experienced a recurrence of disease on ipsilateral pleura after 20 months from surgery and on contralateral lung after 8 months, respectively. Both patients were pT1bN0M0. The first patient underwent chemotherapy and the second one immunotherapy.

**Figure 2.**
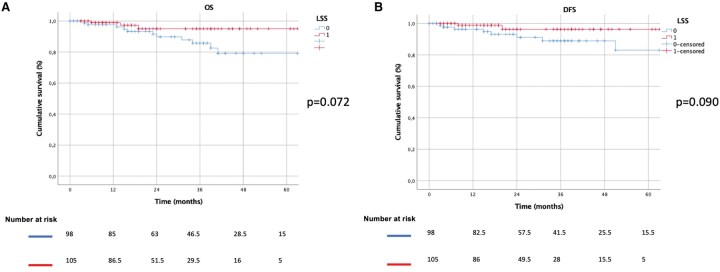
Kaplan-Meier Curves Comparing the Overall (A) and Disease-Free Survival (B) Between LUL and S Groups. Abbreviations: LSS: LUL; DFS: disease-free survival; OS: overall survival

**Figure 3.**
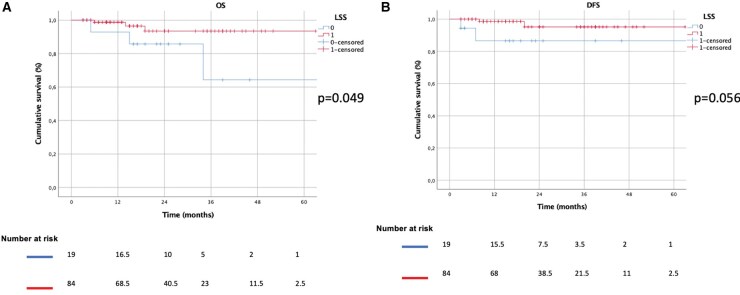
Kaplan-Meier Curves Comparing the Overall (A) and Disease-Free Survival (B) Between LUL and S Groups If Tumour-to-Margin Distance From Surgical Margin (Fissure/Intersegmental Plane) <1 cm. Abbreviations: LSS: LUL; DFS: disease-free survival; OS: overall survival

The median FUP time was 23 months (IQR: 13-42). The 5-year OS and DFS were 95% vs 80% (*P* = .072) and 97% vs 83% (*P* = .090) for LUL and S, respectively (**Figure 2**). Subgroup analyses were also performed, evaluating only cases with tumour-to-margin distance (fissure/intersegmental plane) <1 cm, 5-year OS and DFS were LUL: 98% vs S: 64% (*P* = .049) and LUL: 96% vs S: 87% (*P* = .056), respectively (**Figure 3**). If the distance was >1 cm, 5-year OS and DFS were LUL: 100% vs S: 84%(*P* = .193) and LUL: 100%vs S: 89% (*P* = .351), respectively. In particular, for tumour-diameter >2 cm, the 5-year OS (LUL: 100% vs S: 95%, *P* = .429) and DFS (LUL: 100% vs S: 95%, *P* = .602) were comparable in both groups.

### Multi-segmentectomy cohort

When analysing only the S cohort, no difference was found between lingulectomy and trisegmentectomy in terms of 5-year OS (78% vs 79%, *P* = .240) and DFS (82% vs 84%, *P* = .304). At univariable analysis for prognostic factors affecting OS in S group, no factor reached statistical significance (**Table 3**).

**Table 3.** Univariable Analysis for Prognostic Factors Affecting OS in the Multisegmentectomy Cohort

Variables	Univariable analysis
	*P*-value
Pleural invasion	.306
Tumour-to-margin distance > 1 cm	.159
Tumour dimension >2 cm	.937
Consolidation-to-tumour ratio = 1	.500
pTNM	.556
Positive STAS	.179
N2 LNF retrieved (≥4)	.086
Nodal upstaging	.990
Adjuvant therapy	.713

Abbreviations: OS: overall survival; STAS: spread-through air spaces; LNF: lymphnodes; pTNM: pathologic Tumor-Node-Metastasis staging system.

Univariable analysis for prognostic factors affecting DFS showed pTNM (Pathologic Tumor-Node-Metastasis staging system, *P* = .038) and positive STAS (*P* = .011) as main predictors.

At multivariable analysis, STAS was the only significant predictor for DFS in S group (HR: 0.153, 95% CI (0.029-0.808), *P* = .027), **Table 4**.

**Table 4.** Univariable and Multivariable Analyses for Prognostic Factors Affecting DFS (in Bold Significant Values < 0.05) in the Multisegmentectomy Cohort

Variables	Univariable analysis	Multivariable analysis	
	*P*-value	HR [95% CI]	*P*-value
Pleural invasion	.126		
Tumour-to-margin distance >1 cm	.351		
Tumour dimension >2 cm	.396		
Consolidation-to-tumour ratio = 1	.353		
pTNM	**.038**		
Positive STAS	**.011**	5.088 [0.978-26.471]	**.027**
Nodal upstaging	.518		
Adjuvant therapy	.713		

Abbreviations: DFS: disease-free survival; pTNM: pathologic Tumor-Node-Metastasis staging system;STAS: spread-through air spaces; HR: hazard ratio.

## DISCUSSION

In the last years, the number of segmentectomies has been increasing in clinical practice for the treatment of early-stage NSCLC supported by the publication of the 2 randomized-clinical trials on the topic.^3^^,^^13^ At the same time, a growing interest has been also shown in S, whose role in the treatment of solid tumours compared with the standard lobectomy is still debated.^6–9^

In a meta-analysis^7^ including 5 relevant studies comparing left upper S vs lobectomy for the treatment of early-stage NSCLC, S was associated with reduced in-hospital stay, better 5-year DFS, but similar long-term OS.

A large multi-institutional cohort series conducted in 297 Japanese hospitals,^8^ on a total of 2115 patients who underwent lobectomy or segmentectomy for c-stage I NSCLC in left upper lobe, showed similar postoperative complications, air-leak incidence, and operative duration between the 2 groups.

In our series, a significantly higher incidence (*P* = .014) of minor complications in the S group compared to the LUL group was recorded (**Table 2**), mainly in the number of persistent air-leak (longer than 5 days, *P* = .055). Although an increased incidence of air leakage may adversely influence clinical outcomes—such as prolonged chest tube duration, a higher risk of readmission, and consequently greater healthcare costs—this finding did not translate into a longer hospitalization in the S group, whose length of stay was comparable to that observed after LUL in our cohort.

A plausible explanation for the absence of prolonged in-hospital stay, despite the higher complication rate in patients undergoing S, is the preservation of lung parenchyma, which likely facilitated a more favourable postoperative pulmonary recovery.

Furthermore, intra- and postoperative mortality were null, although some authors considered left apical trisegmentectomy not a simple procedure, with a higher risk of intraoperative and postoperative complications compared with a lobectomy.^2^

In relation to surgical outcomes in our series, the pathological assessment revealed comparable numbers of lymph nodes retrieved between groups for both the total count and total N1 nodes, whereas the number of total N2 nodes was significantly higher in the S group (*P* = .011); even if the nodal upstaging was similar in the 2 groups, the number of total recurrence of disease in S group was significantly higher than in LUL group.

This result could be explained by the subgroup survival analysis performed, where considering patients with a tumour-to-margin distance from surgical margin (fissure or inter-segmental plane) less than 1 cm, 5-year OS was significantly higher in patients who underwent LUL (98% vs 64%, *P* = .049) compared with S, and the 5-year DFS was slightly superior but not significant (96% vs 87%, *P* = .056) in LUL.

Furthermore, it is still unclear what role could play the higher local recurrence rate in long-term survival, considering that parenchymal preservation after segmentectomy may facilitate future radical treatment for local recurrence.

The result of a retrospective non-inferiority study^14^ confirmed our results and showed that DFS following trisegmentectomy for clinical N0 NSCLC in the left upper division are not significantly inferior to those following lobectomy, even if the tumour is located close to the intersegmental plane.

In a more recent study, Liu et al^10^ compared outcomes of left upper bisegmentectomy with LUL, showing that, even in tumours larger than 2 cm, oncological outcomes were similar. In the subgroup analysis of this study,^10^ lingulectomy seemed to have a worse OS than bisegmentectomy and LUL, although no difference was recorded in DFS among the 3 groups.

In our S cohort, no difference was found between lingulectomy and trisegmentectomy in terms of 5-year OS (78% vs 79%, *P* = .240) and 5-year DFS (82% vs 84%, *P* = .304); at uni- and multivariable analyses, only positive STAS was a prognostic factor for DFS, suggesting the need to perform a lobectomy.

Our results are in line with the recent literature also confirming that positive STAS is a negative prognostic factor for unfavourable outcomes and loco-reginal relapse after segmentectomy, suggesting to consider a completion lobectomy.^15^^,^^16^

With the limits of a retrospective study, our results suggest that left upper S can be performed safely, and can be a valid parenchymal-sparing option to lobectomy, not only in case of GGO-dominant but also solid-dominant tumour with a mean diameter of 2 cm or larger, if a surgical margin of more than 1 cm is maintained and STAS is confirmed to be negative.

### Limits and points of strengths

Our study had some limitations that must be considered when interpreting these results. First, the retrospective nature of this study could have influenced the results, so further multi-institutional prospective randomized trials are required in the future; second, selection biases arising from the lack of standardization in the choice of surgical resection (segmentectomy over lobectomy) among different centres, despite the PSM performed, may still exist. These differences are mainly attributable to tumour characteristics (such as pure GGO or dominant-GGO), which can guide the choice of surgical resection but were not available for the analysis, except for the tumour-to-consolidation ratio = 1. Third, surgeon experience may also play a key role in decision-making, often leading to a preference for less risky resections like LUL over S. Fourth, the heterogeneity of surgical approaches used among centres may influence perioperative complications and possibly lymph node yield, although surgical performance of each centre for the adopted technique was of the highest standard. Fifth, intraoperative frozen section was not routinely performed to assess the surgical margin and a centralized pathological review for STAS lacked.

In addition, no data were available regarding histological patterns of adenocarcinoma; longer and standardized FUP protocols among the centres and more details about treatments administered after recurrences were lacking. Furthermore, the relatively small sample size of the S cohort could limit the power of the subgroup analysis of this group.

However, this study had also some points of strength. It was the first multicentre European study on the topic. It involved 9 Italian high-volume centres and reported the most extensive series of upper left S, with the most extended oncological FUP. Moreover, it is the first study comparing also oncological outcomes of lingulectomy vs trisegmentectomy on a multicentre series.

## CONCLUSIONS

S seems to be a safe treatment for early-stage NSCLC located in the left upper lobe, although S seems to expose patients to higher risk of prolonged air-leak in postoperative period, which must be taken into consideration.

However, oncological outcomes after left upper S for early-stage NSCLC are comparable with those of LUL, even in case of tumour larger than 2 cm, if the tumour is located more than 1 cm from the surgical margin. Positive STAS seems to be the only predictor for shorter DFS in S group, confirming the necessity of a completion lobectomy.

## Data Availability

The data underlying this article will be shared on reasonable request to the corresponding author.
